# Inkjet Printing Is a Promising Method of Dyeing Polymer Textile Materials

**DOI:** 10.3390/polym17060756

**Published:** 2025-03-13

**Authors:** Andrey A. Vodyashkin, Mstislav O. Makeev, Pavel A. Mikhalev

**Affiliations:** Bauman Moscow State Technical University, 141005 Moscow, Russia

**Keywords:** textiles, dyeing of materials, polymer, industrial and innovation, inkjet printing, manufacturing industry, textile industry

## Abstract

Inkjet printing is a universal method of direct application and application of various substances to the surface of materials. This technology is gaining popularity in various fields, from textile printing to microelectronics and biomedicine. In the textile industry, inkjet printing has been widely used for many years. In our approach, we systematized the main approaches to maintaining the quality of inkjet printing on various components of materials. We reported and analyzed methods for optimizing the rheological properties of paint to improve the colorimetric characteristics and color fastness on various fabrics. The paper presents surface tension and viscosity regulators, with the help of which the colorimetric indicators of the image on textiles can be improved. For each type of textile, individual modifiers were demonstrated that could most effectively improve the quality of the pattern. Particular attention was paid to the methods of modifying the surface of products, including both physical and chemical approaches. This section discusses an effective method of plasma treatment, which allows you to control the surface free energy for textile polymer materials. By controlling the surface tension of inkjet paints and the surface energy of the material, it is possible to achieve maximum adhesion, thereby significantly increasing the amount of paint per unit area of textile. Additionally, for similar purposes, the principles of chemical modification of the surface with various substances were considered. These methods enable control over the wettability of ink and adhesion to textiles of consistent composition. Additionally, we highlight the potential of thin, optically transparent polymer coatings as a promising strategy to enhance the efficiency of dyeing textile materials. The textile industry is rapidly developing, and the functionality of clothing is improving every year. Inkjet printing methods optimized for maximum accuracy and quality can serve as a significant alternative for applying images.

## 1. Introduction

**Figure 1:**
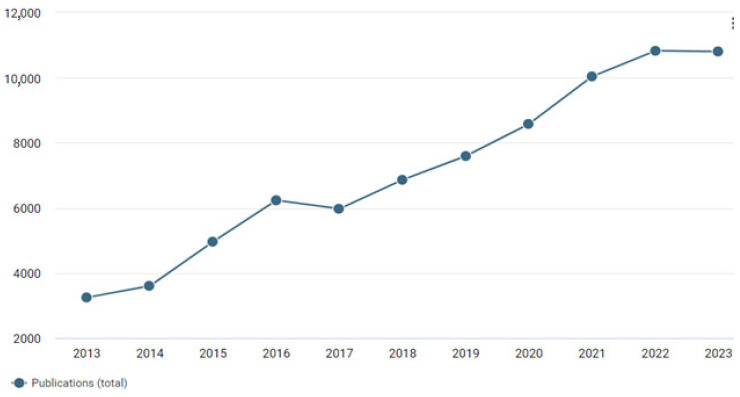
Number of publications in 2013–2023 for the query “inkjet printing” according to Dimensions.

Inkjet printing is increasingly relevant across various fields due to its versatility, which includes the ability to use different types of inks [1,2] and substrates [3], as well as its environmental friendliness, scalability, and technological simplicity [4]. In recent years, interest in inkjet printing has increased due to the possibility of new applications. The number of publications using the term “inkjet printing” has more than tripled in 10 years (Figure 1).

In recent years, inkjet printing has become a popular method for applying prints and images onto various fabrics [5]. This method offers high-quality results, easy scalability, and is environmentally friendly, all at a low cost. Its ability to fine-tune images has made it increasingly popular in the textile industry [4]. Inkjet printing can be used to apply images to polymer materials.

Petroleum-derived polymeric materials have significant potential value in the textile industry due to their low cost and environmental friendliness [6]. Polypropylene (PP) and polyethylene terephthalate (PET) are two examples of such materials that can be produced in large volumes [7]. However, it is important to consider their high surface hydrophobicity, roughness, and heterogeneity when creating final products [8].

The diffusion of ink droplets on textiles is closely related to the clarity of inkjet printing. Diffusion is influenced by the properties of the ink itself, such as viscosity, surface tension, dispersed phase size, and solvent properties [9], as well as the nature and surface properties of the materials [10]. These properties can vary greatly from hydrophobic materials like polypropylene and polyethylene to overly hydrophilic materials like natural fabrics.

Additional surface pretreatments or specific ink additives can be used to improve print quality on a variety of materials. These modifications optimize droplet spreading on the material surface to create high-quality printed products. Techniques currently exist to modify both inks [11] and surfaces [12] to improve color intensity, depth, and color fastness [13].

**Figure 2:**
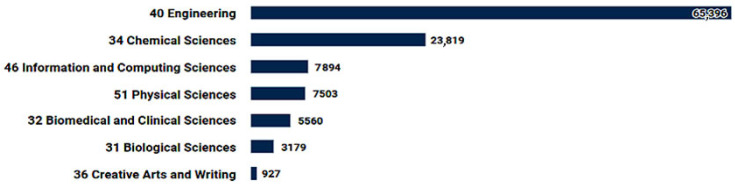
Main publication topics for the query “inkjet printing” in 2013–2023 in the Dimensions database.

Inkjet is of great interest as a method to create hybrid materials that will possess conductive [14], sensitization [15], and biological [16] properties (Figure 2). In addition, direct inkjet printing (deposition) technology is applicable in areas such as ceramics [17], microfluidics [18], photonics [19], optics [20], biological products (incl. organs) [21,22], and surface modification [23,24,25].

In this study, we focus on the reported ways of optimizing ink/surface properties to enhance print quality on textile materials. The review considers various ink additives, including hydrophobic ones, to improve the affinity for surfaces and enhance print quality. Additionally, surface modification techniques for materials that can provide the necessary adhesion to the ink are examined.

## 2. Research Methodology

**Figure 3:**
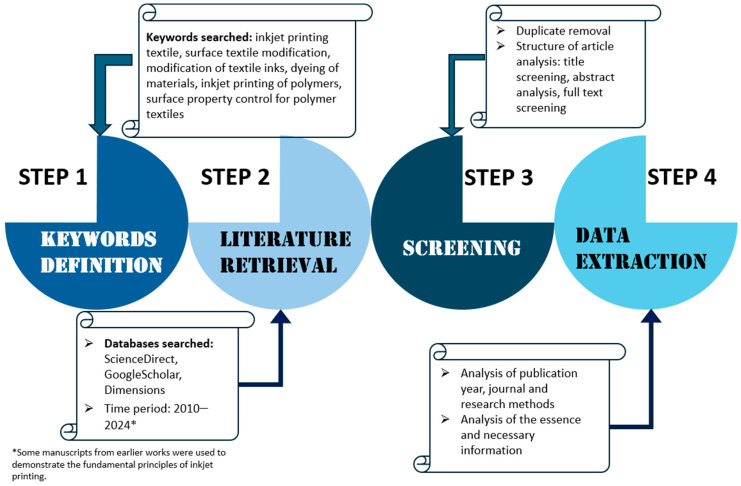
Schematic illustration of our review process.

A systematic literature review procedure was used for this article to examine scientific publications on methods to improve the quality of inkjet printing on textile materials. The search was conducted using ScienceDirect, Google Scholar, and Dimensions. It consisted of searching doe these keywords to find the literature in article titles, abstracts, and keywords. Only English articles in peer-reviewed journals published from 2010 to 2024 were included, although some older and more recent publications were also added to explain the fundamentals of some processes, such as the principles of inkjet printing. Figure 3 shows the detailed process of reviewing this literature.

## 3. Inkjet Basics

Inkjet printing is a technology used to apply dyes or substances to textiles with precise positioning and without contact. It is a cleaner alternative to flat or rotary screen printing, as it avoids fabric contamination. Pigment inks are a promising option for textile inkjet printing due to their short application process, ease of use, energy efficiency, and compatibility with a wide range of textile materials. The improvement of printing quality for pigment inkjet inks has garnered significant attention.

Currently, there are various methods of inkjet printing. A recent review provides a detailed presentation of the different types and methods of inkjet printing [4]. Inkjet printers generate print drops using two mechanisms: drop on demand (DOD) and continuous inkjet printing (CIJ). DOD printing is mainly used for printing graphics and text with relatively smaller droplet diameters (20–50 µm), compared to those of CIJ printing (100 µm). One disadvantage of CIJ is the overconsumption of ink, which can degrade resolution and increase the final cost of the product. In contrast, the DOD process generates individual droplets that meet printing requirements, making it a more economical option than CIJ. There are various types of inkjet printing technologies, including piezoelectric, thermal, acoustic, and aerosol inkjet printing. However, thermal, and piezoelectric are the most used methods. Shah et al. provide a comprehensive review of the classification and basic principles of inkjet printing [4].

### 3.1. Piezoelectric Inkjet Printing (PIJ)

The piezoelectric printhead’s mechanical design comprises an ink chamber that is connected to the ink cartridge through a narrow channel (restrictor/throttle) [26]. At the top of the ink chamber, there is a piezocrystal membrane, typically made of lead-zirconium titanate perovskite that is sandwiched between two electrodes. An electrical pulse is applied to the membrane to eject ink from the printhead. The chamber is pressurized by the membrane, which increases the velocity of the liquid in the nozzle and enables it to be expelled onto an external substrate [27].

Piezo-jet printing technology offers the greatest freedom of ink manifestation compared to any other inkjet technology and has a long head life [28]. However, it is important to note that this technology comes with the disadvantage of higher costs for printheads and associated equipment, which limits its cost-effective integration into low-cost products [29].

### 3.2. Thermal Inkjet Printing (TIJ)

This technology forms droplets by rapidly heating a resistive element in a small chamber containing ink. The printhead comprises a resistor, a chamber, and a nozzle. An electrical pulse applied to the resistor heats the liquid, producing vapor bubbles that push the liquid through the nozzle, thus forming a drop or series of drops [30]. The resistive element’s temperature rises to 350–400 °C, causing a thin film of ink above the heater to vaporize and form a bubble. This bubble creates a pressure pulse that propels a drop of ink through the nozzle [31]. The ejection of the drop leaves a void in the chamber, which is then filled with replacement fluid in preparation for creating the next drop. The resistor is not heated [32]. Thermal inkjet printing has several advantages, such as the ability to produce small droplet sizes and high nozzle densities, resulting in compact devices and lower printhead and product costs. However, it also has limitations on the solvents that can be used. These limitations are the primary disadvantages of thermal inkjet printing. The fluid must have specific boiling point limits and be able to withstand ultra-high temperatures [33].

## 4. Ink for Color Digital Inkjet Printing

Digital inkjet inks have specific indicators and characteristics necessary for successful application to a surface. The properties of inkjet ink vary depending on the type of printing and printer used. Table 1 summarizes the main components of piezoelectric inkjet inks.

**Table 1:** Basic components and performance of ink for digital inkjet printing.

Chemical Composition	Concentration (wt%)								
	[34]	[35]	[36]	[37]	[38]	[39]	[40]	[41]	[42]
Disperse dye	5.01% (magenta 896, yellow 54, blue 359)	4–5% (Carbojet 300)	4.5% (Yellow 54)	3–5% (Blue 359)	3.3% (Red 60)	3% (Blue 359)	3% (Blue 79, Yellow 48)	10% (Red 60)	6% (FW-18 Soot)
Deionized water	64–82%	Balance	68.15–86.15%	61.90–69.90%	48–69%	61.48–65.39%	72.625–73.125%	48.440%	80.70%
Ethylene glycol, EG (C_2_H_4_(OH)_2_)	2.50–22.50%	--	4.50%	23–25%	20.30%	3%	20%	20%	--
Diethylene glycol, DEG ((HOCH_2_CH_2_)_2_O)	5.01%	--	--	--	--	--	--	20%	2%
Sodium naphthalene sulfonate-formaldehyde, MF ((C_10_H_7_NaO_3_S)x(CH_2_O)x)	--	--	--	3–5%	3.30%	3%	3%	15%	--
Organosilicon defoamer	0.50%	--	0.05%	0.05%	0.05%	0.065%	--	0.05%	--
Triethanolamine, TEA ((HO-CH_2_CH_2_)_3_N)	0.05%	--	--	0.05%	0.05%	0.05%	0.05%	0.50%	--
Polyethylene glycol, PEG (g/mol)	--	--	--	0–2%	--	--	PEG1000 2%	PEG400 0–0.97%	--
2-pyrrolidone (C_4_H_7_NO)	--	10%	--	--	--	--	--	--	5%
Glycerol (C_3_H_5_(OH)_3_)	--	--	0–20%	--	1%	--	--	--	4.50%
Polyvinylpyrrolidone, PVP K15, K30, K60 (C_6_H_9_NO)	--	--	0–2%	--	--	--	K30 0.5%	--	
Acetylene glycol derivative, Surfinol 465	--	0.05–0.10%	--	--	--	--	--	--	0.10%
Stirolmaleic anhydride SMA2000 ((C_8_H_8_)n-(C_4_H_2_O_3_)m)	--	0.7–2%	--	--	--	--	--	--	--
Sodium lauryl sulfate, SDS (CH_3_(CH_2_)_11_OSO_3_Na)	--	--	--	--	1%	0.50%	--	--	--
Polyacrylate emulsion ((-CH_2_-CR’(COOR)-)n)	--	--	--	--	0–21%	0–3.906%	--	--	--
Butane-1,2-diol (C_4_H_8_(OH)_2_)	--	--	--	--	--	25%	--	--	--
Propanediol, PGI (C_3_H_6_(OH)_2_)	--	--	--	--	--	--	--	20%	--
Copolymer of polyacrylic acid (Hydrophilic block), TD-1109	2.76%	--	--	--	--	--	--	--	--
Viscosity (mPa⋅s)	1.2–3.2	2.3–2.8	1.81–5.94	2.36–5.11	2.3–11.1	3.8–4.3	2.06–2.56	4.8–9.4	2.73
Surface tension (mN/m)	31–33	30–35	30.14–31.88	34.97–42.01	33.3–34.5	37.8–39	29.78–30.36	42.4–47.6	39.7
Average particlesize (nm)	145.4–166.7 (P), 171.5–186 (W), 171.5–187.5 (C)	--	183–198	142.6–152.8	131.3–147.8	167–170.5	184.9–188.4	165.5–165.3	163.4

### 4.1. Characterization of Dispersed Inkjet Inks

Inkjet inks must be formulated carefully to ensure optimal droplet formation within the fine nozzles of the printhead for high-speed application. This should be followed by optimum surface spreading and high color performance.

Aggregation of dye molecules is a significant issue in inkjet printing. It can cause uneven distribution of printing ink on the fabric surface, affecting printing accuracy. Therefore, it is crucial to consider the effect of inkjet ink composition on droplet formation, flight path, and substrate surface morphology [43]. Color inkjet inks typically contain dyes [44], organic solvents [45], defoamers [46], pH adjusters [42], and viscosity adjusters.

**Figure 4:**
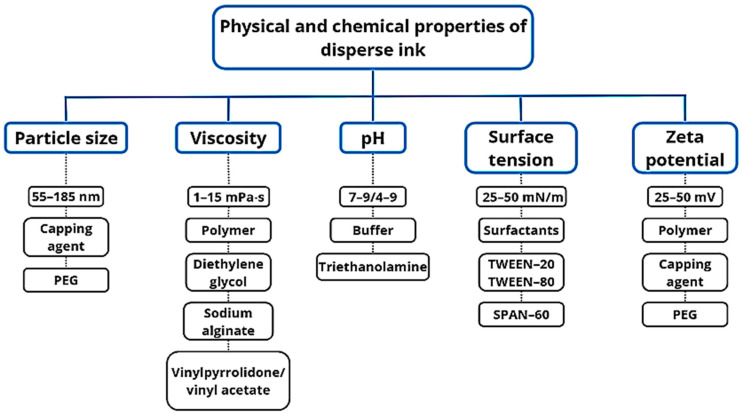
Physicochemical properties of standard disperse inks.

Figure 4 displays the primary physicochemical properties of dispersed inks that impact textile printing quality. Viscosity is a crucial factor in achieving favorable inkjet printing outcomes as it affects filament stretching (droplets/droplet satellites) and fluid behavior during the process. Surfactants decrease ink surface tension and guarantee consistent drop ejection from the inkjet printer head nozzle. To optimize the formulation, it is important to pay close attention to the nature and amount of chemicals due to the binding affinity between surfactants and colorants. Consider the structure of surfactants and dyes, critical micelle formation concentration, maximum additive concentration, and other relevant parameters [47,48].

The viscosity, surface tension, particle size, conductivity, zeta potential, and distribution of ink are all significantly impacted by the presence of surfactants and moisture-binding agents in inks and pigments. Printing quality is influenced by several key factors, including the speed of droplet formation, the impact and spreading of droplets on the substrate, substrate roughness, and ink dispersion.

**Table 2:** Physical property values for standard disperse inks used in various inkjet printers.

	Viscosity, mPa⋅s	Surface Tension, mN/m	Particle Size, nm	pH	Zeta-Potential of Particles, mV	Reference
Epson R330 (Seiko Epson Corporation, Japan)	1.6–11.1	33.2–37.3	112.3–147.8	7.6–8	25–35	[41,48]
Dimatix DMP-2800	2.8–3.2	30–49	129–160	7–9	–	
HP Deskjet	2.1–3.5	26–38	41–89	10	34–55	[49]
RK Flexiproof 100 (TMI Machines, USA)	1.93–5.59	30–32	145–190	7–8	–	[50]
Epson R230 (Seiko Epson Corporation, Japan)	2.06–3.24	31.09–32.95	147.2–166.7	7.6–7.8	–	[36]

By varying the parameters of physicochemical parameters of dispersed inks such as viscosity, surface tension, particle size, pH and zeta potential, problems related to line doubling, line breaking, flowability and spattering in inkjet printing on fabric fibers can be solved (Table 2) [51].

### 4.2. Dispersed Inks for Dyeing Textile Materials

#### 4.2.1. Inkjet Ink for Printing on Nylon Fiber

Nylon is a synthetic polyamide widely used in fiber manufacturing due to its advantageous properties, including heat retention, comfort, and practicality [52]. However, it is important to account for its limitations. At elevated temperatures (approximately 100 °C and above), the fiber structure can deteriorate, resulting in decreased durability and a loss of aesthetic appeal. Furthermore, nylon is not ideal for wear in hot weather, as it lacks the sweat-absorbing properties of natural fabrics, potentially causing skin irritation [53].

Nylon fibers are utilized across a broad range of applications, such as the production of backpacks, hiking and sports equipment, carpets, and more [54]. Digital printing on nylon substrates has recently gained popularity due to its versatility and precision. A critical factor in achieving high-quality digital prints on nylon fibers is the selection and application of an appropriate thickener in the printing ink. Digital printing requires significant amounts of ink, necessitating the use of cost-effective and efficient thickeners. The thickeners listed in Table 3, commonly used in inkjet printing, must demonstrate stability in the printing paste and maintain an acceptable viscosity.

**Table 3:** Indicators for various thickeners as additives in pigment ink.

Thickener	Concentration, %	Viscosity, mPa⋅s	Surface Tension, mN/m	Reference
Sodium alginate (SA)	0.5–1.75	0.18–4	31–36	[55]
Natrozole	3	3–20	32–36.3	[56]
Polyacrylic acid	1	0.18–4.00	29–35	[56]

Abd El-Wahab et al. identified sodium alginate (SA) as the optimal thickener for disperse inks, primarily due to its ability to regulate viscosity. Adjusting binder concentration influences both viscosity and surface tension, directly affecting ink spreadability. The study noted that viscosity levels ranging from 3 to 28 mPa⋅s exceeded optimal parameters for disperse inks. Inks with viscosities above 15 mPa⋅s were unsuitable for use due to poor flowability during ejection from printhead nozzles, while inks with viscosities below 11 mPa⋅s were more effective for printing.

The balance between viscosity and surface tension—affected by substrate properties like porosity—was critical for achieving deep ink penetration. A surface tension range of 31–36 mN/m enabled penetration depths of 90–100%. Maintaining a pH of 4–5 during ink formulation was essential for ensuring thorough ink absorption into nylon substrates [54].

**Figure 5:**
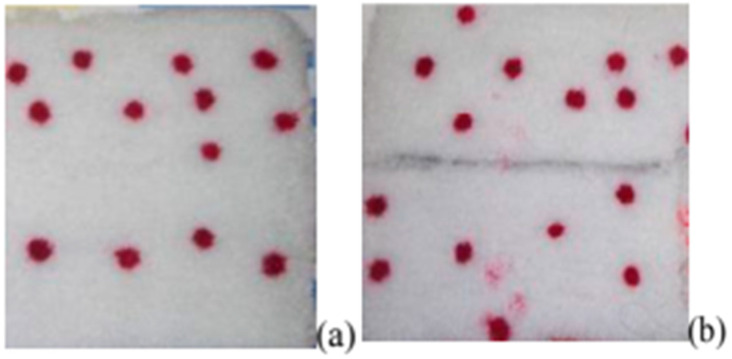
Image of circles printed on carpet materials using different ink additives: (**a**) Natrosol, (**b**) sodium alginate [54].

The addition of thickeners enables precise control of viscosity and surface tension in inkjet ink formulations for nylon fibers. As shown in Figure 4, prints on nylon fabrics and carpets using ink with thickeners exhibit greater optical density compared to inks without added binders. By optimizing the type and concentration of surfactants, print quality can be significantly enhanced, ensuring better ink flow and coverage on the material surface (Figure 5) [54].

#### 4.2.2. Inkjet Ink for Printing on Cotton Fiber

Cotton fabric is a material obtained by modifying cotton with fibers of other origin, such as polyester or viscose, among others. The manufacturing process of such fabrics involves the use of different yarn knitting methods, which gives them a variety of looks and characteristics. A single cotton fiber resembles a fine hair, measuring between 15 and 25 microns in diameter and ranging from 6 to 52 mm in length. Under microscopic examination, it appears as a twisted, turned, and flattened tube-like structure [9].

There are some disadvantages to using cotton fabric. Firstly, it is easy to wrinkle, and it is difficult to smooth out the wrinkles afterwards. Second, it is also prone to shrinkage, and the degree of shrinkage can range from 4% to 10%. Third, cotton fabric is not acid resistant. If low pH substances such as vinegar or other acidic substances get on such fabric and are not removed in time, they may cause irreversible damage [57].

Cotton fabrics can be printed with inks based on reactive dyes. Reactive dyes are special dyes that form a chemical bond with the fiber, penetrating its structure and becoming an integral part of it. This results in durable and long-lasting colored materials. Commercially available reactive inkjet inks typically use dyes with low to moderate tack, so maximum dye fixation to the fabric is important for economic and environmental reasons. In the application process, reactive dyes are typically applied to cotton fabrics in the presence of alkali to allow the dye to bond to the fabric by forming a covalent bond between cellulose anions and reactive groups in the reactive dye molecule [58]. Table 4 describes some of the additives that can be used in the formulation of reactive inks for printing on cotton [58].

To prepare the reactive ink, Lei Wang et al. conducted a study which showed that pretreatment of cotton fabric with alkali and urea had a positive effect on the binding of the ink to the substrate, and the dye fixation rate was 89.13 s. Tween-80 surfactant, an emulsifier responsible for ink consistency, viscosity, and plasticity, was used as an additive in the ink. The viscosity and surface tension parameters of the reactive ink were 3.7 mPa⋅s and 41.79 mN/m, respectively [59]. For deep penetration of the ink into the cotton fabric substrate, the acidity of the medium was pH 7, and the absorbance of the fabric ink was 70% [60].

**Table 4:** Characteristics of additives in inkjet ink for printing on cotton fibers by inkjet printing.

Additive	Concentration, %	Viscosity, mPa⋅s	Surface Tension, mN/m	Reference
N-methylmorpholine N-oxide	2	2–2.8	28–31	[57]
Diethylene glycol	0–50	1–6	57–75	[61,62]
Carboxymethyl cellulose	3	3.35	27–34	[63]

Hui Peng et al. also showed that increasing the concentration of DEG diethylene glycol had a positive effect on the color fastness and clarity of the resulting printed images. The concentration of DEG was varied from 0% to 50% by weight. As the concentration of DEG increased, the viscosity of the ink gradually increased due to the molecular structure of each component in the ink composition. The van der Waals forces between the molecules increased, so the viscosity of the system increased from 1.1 to 6.2 mPa⋅s. The surface tension of the paint was in the range of 57–76 mN/m. The hydrophobic carbon chain of DEG leads to a decrease in the surface tension of the ink. When 10% DEG was added, maximum clarity and colorfulness of the printed patterns was achieved [61,62].

**Figure 6:**
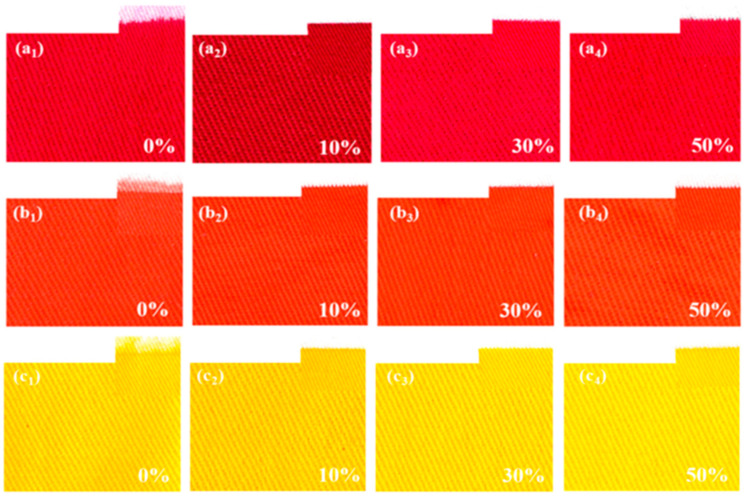
Effect of different DEG concentrations on print clarity and color intensity, reproduced from [9].

When printing on cotton fabric, using an emulsifier and DEG during the preparation of reactive ink, a concentration of 0.25% by weight of Twin-80 and a mixture of 10% DEG dye solution resulted in the highest indicators of printing clarity of drawings (Figure 6), K/S index (color intensity), and the highest contrast and saturated colors. Additionally, additives can be used to improve the printing properties of reactive dye solutions on cotton fabrics in an effective and environmentally safe manner.

#### 4.2.3. Inkjet Ink for Printing on Silk

Silk is a delicate fabric known for its soft texture, flexibility, and lustrous appearance. It is produced from threads spun from the cocoons of silkworms, specifically the mulberry silk variety. Each thread can extend up to 1.5 km in length and has an average thickness of 30–40 microns. The fabric’s characteristic shimmering effect is due to its unique cross-sectional geometry, which causes light to refract in distinct ways. Notable qualities of natural silk include its ability to regulate temperature, its moisture permeability, as well as its hypoallergenic and bactericidal properties, and resistance to abrasion. However, silk is often costly [64]. Despite its luxurious attributes, silk also has its drawbacks, such as its tendency to wrinkle easily and the formation of streaks or stains even from minor moisture exposure [65].

For organic pigments to be effectively applied to silk fabric, they require surface modification. Currently, challenges include issues with concentration gradient formation, jet blockage, color inconsistency, and low optical density in the resulting images. It is crucial that pigment dispersion maintains colloidal stability, with controlled particle size growth, ensuring that the particles remain below 185 nm over time [66].

**Figure 7:**
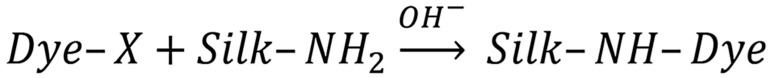
Scheme of the side interaction of a reactive dye with silk fiber.

Polysaccharide-based thickeners, such as highly substituted hydroxypropylcellulose (H-HPC), sodium alginate (SA), hydroxyethylcellulose (HEC) [67], and guar gum (SG-9), have been proposed as binders. The concentration of these thickeners ranges from 0.5% to 3%. The viscosities of these thickeners are as follows: H-HPC at 0.74 mPa⋅s, SA at 1.02 mPa⋅s, HEC at 0.66 mPa⋅s, and SG-9 at 0.11 mPa⋅s. The contact angles of the mixed thickeners were also measured. The contact angles for H-HPC/SA, H-HPC/SG-9, and H-HPC/HEC were 59.6°, 63.9°, and 63.0°, respectively. The H-HPC/SA thickener showed poor printing clarity on fiber due to its low wetting contact angle of 59.6°, suggesting rapid ink absorption and potential excessive ink migration after printing, possibly due to the presence of auxiliaries or impurities in the SA. Nevertheless, the adhesion properties of H-HPC/SA can help minimize side reactions (Figure 7) with the reactive dye, thus enhancing the color rendering of the printed pattern [68].

Untreated silk fabrics can be printed with reactive dyes using inkjet technology. The adhesion process between the dye and substrate fixes the reactive ink. The depth of dye penetration into the substrate depends on the amount of H-HPC thickener added and the chemical nature of the secondary polysaccharide thickener. The prints produced with H-HPC/SG and H-HPC/HEC showed clear and fairly uniform sharpness. However, the use of H-HPC/SA resulted in a deterioration of the sharpness of the printed pattern [65].

#### 4.2.4. Inkjet Ink for Printing on Polyester Fiber

Polyester, also known as polyethylene terephthalate (PET), is a synthetic fabric that resembles cotton or wool. Its production technology results in a material with high strength, excellent elasticity, and the ability to retain its shape well [69]. Polyester is a popular material in the textile and fashion industry due to its advantageous properties. It is also highly valued for its quick-drying ability, making it an ideal choice for sports and activewear. Moreover, polyester is long-lasting and low-maintenance, making it a popular choice for bed linens, curtains, pillows, and other home furnishings [70].

Polyester has several disadvantages to consider when choosing a material for clothing or other items. Firstly, it is prone to electrification, which can cause dust and other fabrics to stick to it. Secondly, it is not breathable and is not recommended for use in hot weather. Additionally, it can be challenging to remove tough stains from polyester. Finally, it should be noted that polyester is highly flammable and can cause fires. Pure polyester is also quite stiff and can cause skin chafing when worn. Polyester fibers are primarily dyed with dispersed dyes due to their high surface charge. However, the dyeing process can be slow under standard conditions due to the highly crystalline and hydrophobic nature of polyester, even when small molecule size dyes are used [71].

Polyester inks use a water-dispersible polymer that promotes the formation of hydrated polymer mesh structures. In various industrial applications, including tissue engineering, drug delivery systems, medical diagnostics, and implant coating, NVP/VA 64 (polyvinylpyrrolidone) polymer has found widespread use. In textile printing, NVP/VA 64 has good compatibility with many organic dyes. It is commonly used to blend with hydrophobic synthetic fibers, such as polyacrylonitrile, esters, nylon, and other fiber materials, to enhance their dyeability and hydrophilicity [41,72]. Chengyong Gao et al. demonstrated that the addition of NVP/VA copolymer (NVP/VA64) to disperse dyes improves the quality and clarity of the printed pattern, resulting in a particle size of 190–200 nm that allows for inkjet printing. The viscosity of the resulting printing ink ranged from 2.3 to 11.1 mPa⋅s. As viscosity increased, the ink’s passage through the printhead nozzle deteriorated and the relaxation time increased. Therefore, the optimum viscosity for the least ink spreading on the surface was 3.2 mPa⋅s. The ink’s surface tension was 33.2–35.8 mN⋅m^−1^, at which point it was able to wet the polyester fibers. To achieve a clear print line, the surface tension needed to be 35.5 mN⋅m^−1^ [38].

In their subsequent work, Chengyong Gao et al. proposed the use of polyacrylate emulsion as an additive. The concentration of the additive in the ink ranged from 0% to 3.91% by weight. The zeta potential of the prepared disperse ink ranged from 47 to 53 mV, indicating that the disperse dye in the ink did not agglomerate and had good stability. The indelible disperse ink met the requirements for printing, with an average particle size of about 167.5 nm. The viscosity of the ink ranged from 3.8 to 4.3 mPa⋅s, which can help to improve the clarity of the printed pattern by attenuating the diffusion of ink over the surface. The surface tension was 38.0–39.0 mN/m. The standard parameters for ensuring high contrast, sharpness, and low line spreading of the printed pattern were determined to be viscosity (3.9 mPa⋅s), transverse relaxation time (413 ms), and surface tension (39.0 mN/m) [39]. NVP/VA can be used as a carrier for disperse dyes to improve solubility and film formation on the surface of printed fabrics, which helps to improve color fastness on polyester fibers.

**Figure 8:**
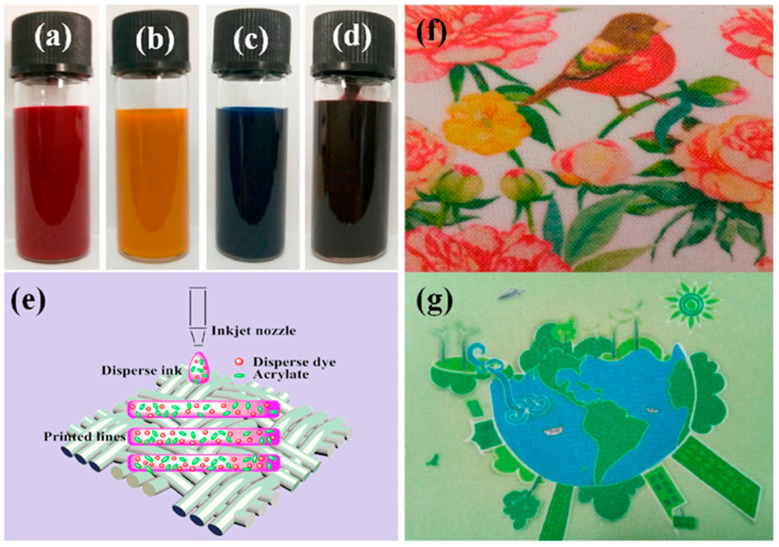
Samples of modified dispersion ink (**a**–**d**), inkjet dispersion ink on polyester fabric (**e**), and print sample on polyester fabric (**f**,**g**), reproduced from [38].

The indelible disperse inks contain a polyacrylate emulsion that aids in fixing the disperse dyes to the fabric surface. This process involves thermofixing the dispersed dye to the fabric surface, which results in the formation of a polyurethane layer on the printed pattern. The polyurethane film enhances the color fastness of the printed fabric, as demonstrated in Figure 8 [38].

#### 4.2.5. Inkjet Ink for Printing on Polypropylene Fiber

Polypropylene is a type of polyolefin, which is a high molecular weight hydrocarbon of the aliphatic series. Compared to polyamide fiber, polypropylene fiber has several advantages, including greater resistance to double bends and higher elasticity. Additionally, it has a melting point of 135 °C and ignites between 325 and 385 °C. The density of polypropylene fiber can vary widely. Polypropylene is a cost-effective material derived from hydrocarbons that simplifies manufacturing processes. Its properties make it suitable for various applications [73,74].

Polypropylene is a partially crystalline, non-polar plastic and the second most widely used plastic globally. Its significance is growing in the production of various textile materials, particularly in the development and manufacture of personal protective equipment [75,76]. Researchers have attempted to enhance the color of pigment-based inks with conventional binders using various methods, including the use of amino compounds, chitosan and its derivatives, ß-cyclodextrin, citric acid, ink coagulants, and acrylic resin to anchor the ink to the substrate. Similar compositions can be used as inkjet printing ink base. However, the use of pretreatment components and commercial textile binders in ink compositions can lead to the deposition of adsorbed layers that are thicker and stiffer. Furthermore, the physical properties of pigmented inkjet-printed fabrics can be negatively impacted by high-temperature curing of pretreatment components and subsequent higher curing temperatures [49]. Monomers containing terpene acrylic linkages enhance the adhesion of polypropylene and increase its resistance to surface oxidation. Acrylic polymers with terpene functional groups exhibit exceptional adhesion to surfaces with low surface energy and large edge angles [77].

Osama et al. demonstrated that a binder containing aminopropyl/vinyl/silsesquioxane (APSV) can be used as an additive in disperse inks. Dispersed inks containing APSV binder (0.25–0.75%) and other polymers exhibit stable particle size, surface tension, and viscosity. The encapsulating cationic shell of APSV reduces flocculation of pigment particles, allowing the dispersed ink to be used at low viscosity values. The viscosity of the inks ranged from 2.85 to 4 mPa⋅s. Increasing APSV concentration resulted in a decrease in particle size, with an average particle size ranging from 110 to 150 nm. The zeta potential ranged from 34.6 to 52 mV. The contact angle with water ranged from 64.1° to 84.0°. The ink containing APSV had a strong affinity for polypropylene due to the low surface energy of the fiber and dispersed ink. The polymer contains silicone compounds that can reduce the surface tension of any material and act as effective wetting agents. The surface tension was adjusted to a range of 30.0–38.0 mN/m [49]. APSV enhances the adhesion of pigment-based inkjet inks to polypropylene fiber and can significantly improve color performance and degree of fixation (DF percentage) compared to commercial textile binders. Additionally, the APSV polymer exhibits strong antistatic properties. Pigment colors are effectively fixed on polypropylene at low stripping temperatures through thermal initiation or ultraviolet (UV) irradiation of vinyl end groups in APSV.

This section demonstrates that optimizing the physicochemical parameters of the ink can increase the efficiency and improve the color characteristics of textile materials dyed using inkjet printing. Specific additives can be used for certain materials to improve the functional properties of the ink, adhesion to the surface, and spreading on the surface. Such substances may include thickeners, pH regulators, and surfactants. It is important to optimize the ink properties for each type of material used for printing. This optimization provides improved color and resistance of images to various influences.

## 5. Modification of Material Surfaces

Certain materials commonly used in the textile industry possess characteristics that restrict the use of standard inkjet printing methods. These materials may have poor color rendering, low ink adhesion, or a surface that is too hydrophilic due to their nature and manufacturing process. As a result, pretreatment of the fabric is necessary to enhance the outcome of inkjet printing. Materials with low surface energy, such as polypropylene (PP) and polyethylene (PE), are noteworthy. According to Young’s equation, the wettability of ink on a surface is determined by the relationship between the surface energy of the substrate and the surface tension of the ink. For optimal ink spreading on the material, the surface energy must be higher than the ink surface tension. It is important to note that surface type, as well as macro- and micro-relief, can have additional effects that need to be considered when printing [78]. According to Park et al., the most significant factors for textile printing quality are fabric structure, yarn size, and the hydrophilic/hydrophobic nature of the fabric [79].

To enhance the surface free energy, the material can undergo pretreatment, which may involve physical or physicochemical actions, or coating with various substances. Fabric pretreatment is essential to achieving good color fastness, excellent resistance to satellite droplet formation, control of ink spread penetration on the surface, and optimal image quality when digitally printing on textiles [80].

### 5.1. Physical Methods of Surface Modification

Modern methods of hydrophilization and washing of materials often require significant time, energy consumption, and the use of special reagents. Unfortunately, these methods can also lead to the formation of toxic substances and wastewater, which can cause serious environmental problems. Many modification options use extreme modes for fabrics, such as heating in an alkaline bath, which can significantly deteriorate the strength properties of materials. It is important to note that even with intensive exposure, the surface may not acquire the necessary hydrophilic properties, which can hinder the spread of ink and worsen printing.

**Figure 9:**
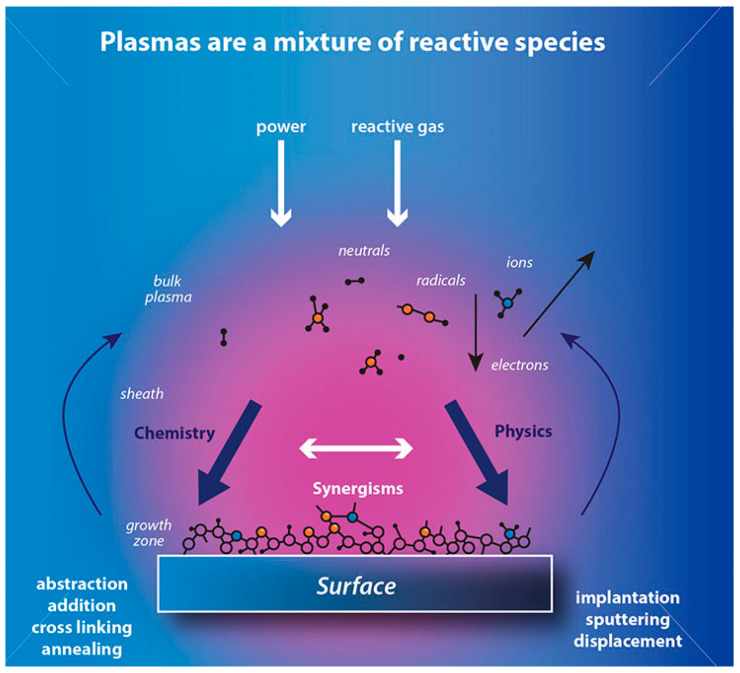
Mechanism of plasma surface treatment, reproduced from [83].

Modern techniques such as plasma treatment (Figure 9), knot treatment [81], and microwave exposure can improve processing efficiency, clean surfaces, hydrophilize, and alter surface chemical composition. Research data indicate that low-pressure plasma treatment can achieve up to 90% energy efficiency, depending on volume, target, ink type, and exposure time. However, the implementation of alternative methods that conserve water, chemicals, and energy requires significant initial investment and extensive research before commercialization. Thus, the use of advanced high-tech methods is currently restricted [82].

**Figure 10:**
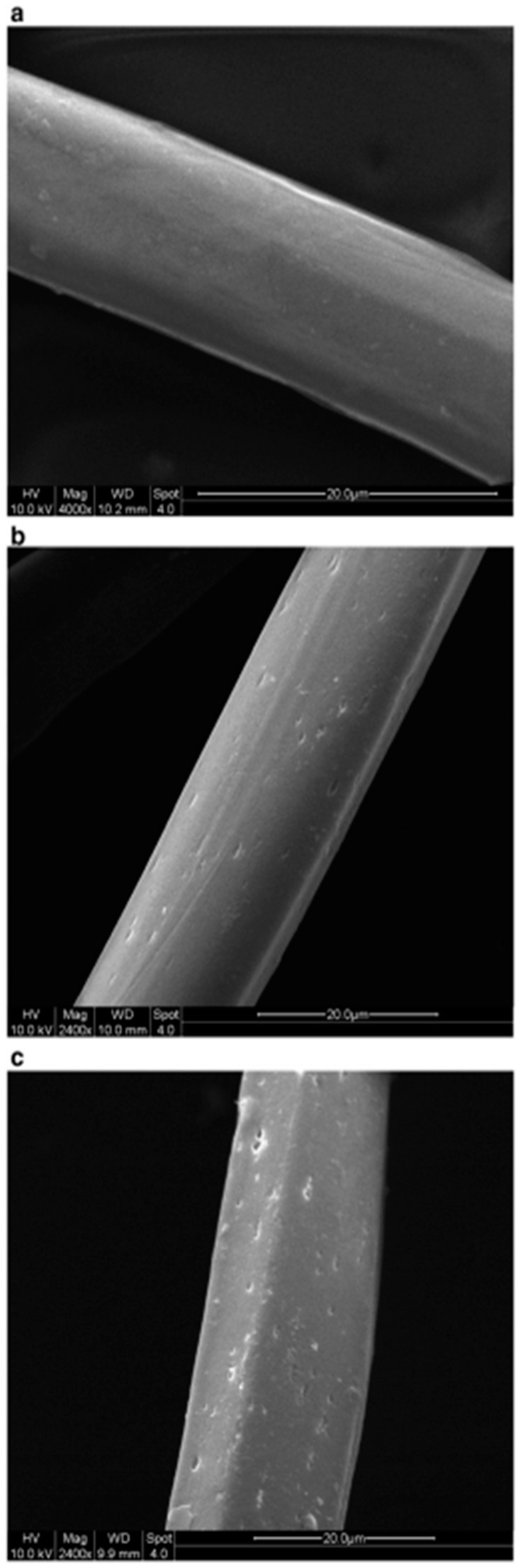
SEM images of polyester fibers: (**a**) untreated, (**b**) treated with air plasma, and (**c**) treated with argon plasma, reproduced from [84].

Zhang and Fang proposed a method for surface modification of polyester fabric through plasma treatment with air containing 10% argon (Figure 10). The change in surface morphology was confirmed using electron microscopy, which is related to the plasma etching of the fabric surface. X-ray phase analysis was used to confirm the activation of the surface and the enhancement of the O1s signal relative to the untreated sample, as well as the appearance of the N1s peak. The plasma treatment had a significant effect on the surface properties of the material. Wetting edge angle analysis confirmed a marked increase in surface hydrophilicity. Additionally, the plasma treatment improved the quality of inkjet printing, specifically increasing color and saturation while slightly decreasing brightness. However, the treatment did not affect the ink’s resistance to mechanical stress [84].

Pransilp et al. also applied plasma treatment to improve surface properties for inkjet printing on cotton. The study employed O_2_, N_2_, and SF_6_ gases for plasma treatment. Atomic force microscopy confirmed that atmospheric and oxygen plasma caused surface disruption, resulting in a significant increase in surface roughness. In contrast, nitrogen plasma treatment had no significant effect on surface roughness. The findings were confirmed by scanning electron microscopy, which revealed cracks and scratches on the surface. The wetting angle decreased with oxygen and nitrogen plasma treatment, depending on the intensity and duration of the treatment. Conversely, treatment with elevated gas increased the edge angle up to 115 degrees, which was attributed to the fluorination of the surface groups of cotton. During the test printing with aqueous ink, it was observed that the samples modified by oxygen plasma produced the brightest and most colorful images. This is due to the increase in surface free energy and the formation of new bonds on the material’s surface. However, it should be noted that intensive plasma treatment resulted in a decrease in fabric mass, which in turn reduced the material’s strength properties [85].

**Figure 11:**
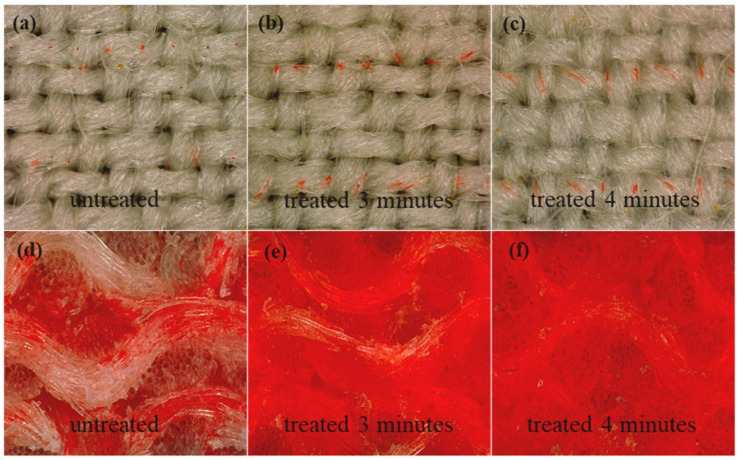
Distribution of reactive dye droplets on the surface of wool fabric (**a**–**c**); cross sections of wool fabric for different times of atmospheric plasma treatment (**d**–**f**), reproduced from [86].

Thakker et al. used air plasma to modify the surface properties of cotton and wool materials for inkjet printing. The treatment resulted in surface degradation and a significant reduction in the marginal wetting angle for each material. The researchers then used plasma modification to print with inks derived from plant extracts. The print quality of the cotton samples improved significantly in terms of brightness and color, compared to the untreated materials (Figure 11) [6].

In Wang et al.’s work, untreated raw wool (480 g/m^2^) was subjected to air plasma treatment for 1 to 4 min [86]. X-ray photoelectron spectroscopy (XPS) confirmed an increase in hydrophilic groups, such as C=O (288.0 eV), C-O/C-N (285.7 eV), and C-C/C-H (284.8 eV), on the material’s surface. Furthermore, the plasma treatment resulted in an increase in surface hydrophilicity and droplet absorption rate into the wool. When using reactive dyes for inkjet printing, longer processing times led to higher dye concentrations on the material, resulting in improved pattern quality. Additionally, plasma-treated wool samples exhibited enhanced abrasion resistance and washability, attributed to the higher dye concentration per unit area of the material [86].

In addition to plasma treatment, topical ultrasound treatments can also improve tissue performance. Ultrasound is a sound wave that creates microbubbles in the solution through the cavitation phenomenon. This phenomenon creates micro-jets and shock waves that facilitate micro-stirring, mixing, and mass transfer in a chemical reaction [87]. The combined use of ultrasonic treatment with various chemicals can increase the effectiveness of the treatment. Cavitation creates bubbles that collapse at high velocity, producing locally critical temperatures and pressures near the microbubbles.

Körlü and Bahtiyari’s work presents a comprehensive review of the possibilities and options for processing various textile materials using ultrasound [88].

Gotoh et al. demonstrate a highly efficient process for cleaning various material surfaces through shaking and ultrasonic treatment. These methods can be used for pretreatment and surface activation before inkjet printing [89].

We aim to also address the lack of research into the scale-up and modernization of industrial textile facilities, particularly in the area of textile inkjet printing. In their study, Perincek et al. demonstrated the potential for creating an industrial bath for ultrasonic processing and optimized the bath parameters for uniform acoustic pressure. Additionally, this study examined the impact of tissue parameters and irradiation intensity on the final exposure effect. The results confirmed that the characteristics of the tissue have a significant influence on the effect of ultrasound exposure. Therefore, it is necessary to personalize such setups for each type of material [90]. This work represents a crucial step in demonstrating the possibility of optimizing the effect of ultrasound on tissues on an industrial scale. Thanks to this work, the transfer of fundamental methods and technologies can be significantly accelerated, and the efficiency of technological processes can increase.

Microwave radiation has been used for textile improvement processes since the mid-20th century. During this time, cellulose fabrics were treated with Durable Press finishing agents and cured with microwave radiation [91].

Additionally, Narendra V. Bhat et al. utilized microwave radiation to enhance the performance of polyester textile yarns. The exposure was conducted using a microwave oven operating at a frequency of 2450 MHz and a power of 850 W. As a result of this exposure, the dye’s absorption capacity increased threefold. This phenomenon can be attributed to molecular rearrangements, which were confirmed by X-ray analysis. This method combines the synergistic effects of microwave waves and fiber heating, which together allow for the alteration of the polymer’s macromolecular structure. The DSC analysis indicates a higher rate of crystallization and a narrower distribution, suggesting a high degree of conversion during the fiber modification process [92].

### 5.2. Chemical Methods of Surface Modification

One traditional method for surface modification during pretreatment is coating the surface with a thin film made of various polymers. Polysaccharides, such as sodium alginate, are commonly used as modifying agents [93,94].

**Figure 12:**
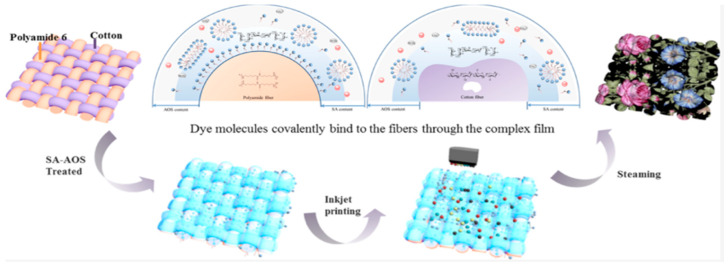
Tissue surface modifications with sodium alginate and alpha oleinsulfonate, reproduced by [95].

In a study by Chang Li and colleagues, sodium alginate (SA) and alpha oleinsulfonate (AOS) were used as a modifying coating for woven cotton and polyamide fabrics (Figure 11). The authors discovered that the optimal concentration of sodium alginate for all colors is 2%, while the optimal concentration of AOS is 5%. Beyond these values, the intensity of the printed images did not increase. Electron microscopy techniques revealed that the addition of AOS to the SA structure helped to organize and structure the film, resulting in a rough structure with tubercles. X-ray techniques confirmed successful film formation on the material’s surface. The analysis of printed images confirmed the synergistic improvement of homochromaticity in cotton and polyamide, resulting in the formation of the “deepest” color. This was due to the improved distribution of ink on the material’s surface (Figure 12) [95].

An et al. discuss the effects of enzymes and sodium alginate on wool for inkjet printing [96]. Liang et al. improved printing quality and increased fixation rate on the surface of linen fabric by using hydroxyethyl cellulose, alkali, and urea for surface modification [97].

Zhao et al. used an alginate propylene glycol PGA-based modifying compound in combination with urea and sodium hydrogen carbonate to enhance the quality of inkjet printing on cotton. The polymer solution increased the edge wetting angle from 50° to 70° and smoothed the surface, as observed in the SEM images. The PGA modified samples exhibited the best color depth and saturation due to the good hydrophilicity and moisture retention of the PGA film, which facilitated controlled ink spreading on the surface [98].

**Figure 13:**
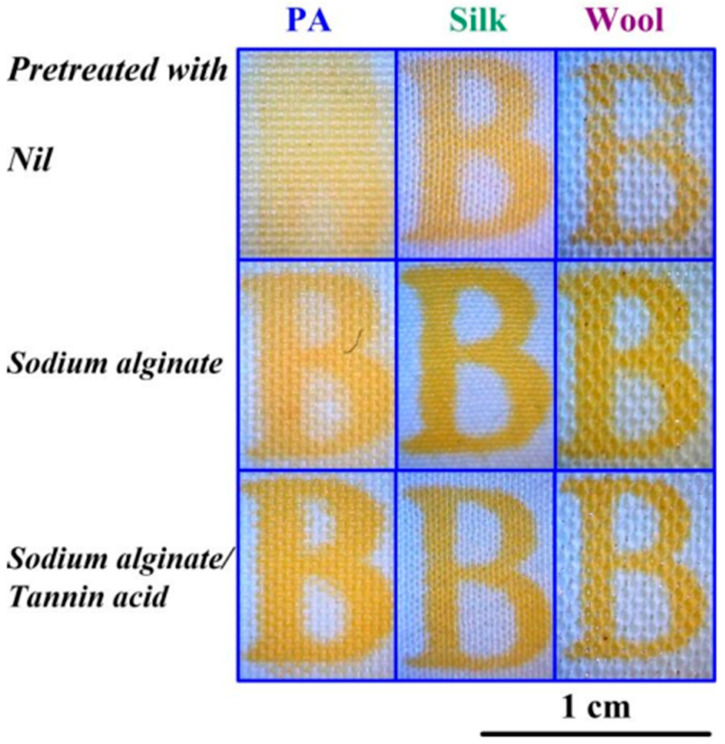
Fabric samples with various pretreatments, reproduced from [99].

Zhou et al. presented methods for surface modification of polyamide (PA), wool, and silk fabrics to enable curcumin dyeing via inkjet printing. The authors used sodium alginate (SA) and a mixture of SA and tannic acid as modifying compositions (see Figure 13). A piezoelectric printer (Dimatix Sapphire QS-256/80 AAA) was used to print on these materials with inks containing curcumin and surfactants. The surfactants acted as surface tension regulators and promoted the transfer of curcumin into solution. The study confirmed an increase in the hydrophilic properties of the surfaces of all materials, resulting in deeper pigment penetration into the fabric. The modified samples exhibited higher resistance to mechanical stress. Additionally, those treated with tannic acid showed improved lightfastness compared to the other samples. It is important to note that the tannic acid pretreated samples demonstrated antibacterial properties against E. coli, although not as effective as the curcumin-based ink coated samples [99].

**Figure 14:**
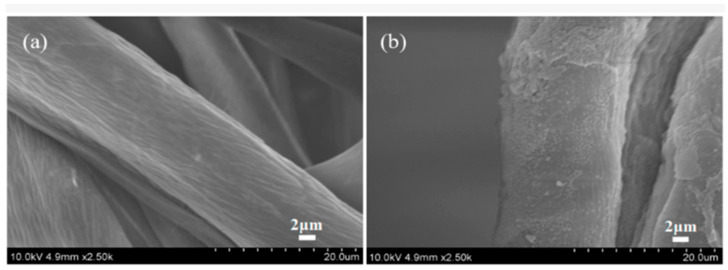
Cotton fibers: (**a**) before treatment, (**b**) after treatment; reproduced from [100].

Yang et al. presented the possibility of modifying cotton using organic nanospheres and sodium hydrogen carbonate. They obtained hybrid particles by co-polymerizing styrene, butyl acrylate, and vinylbenzyltrimethylammonium in a nitrogen atmosphere at 80 °C. The modifying particles’ nanoparticles had a broad distribution with an average size of approximately 65 nm, as measured by SEM. In inkjet printing, the image intensity showed a concentration dependence on nanoparticles in the range of 0 to 1 g/L. However, further increase in concentration did not improve image quality. This effect is attributed to the hydrophilic nature of the ink, which can bind to both hydroxyl groups on the cotton surface and quaternary ammonium groups of the nanospheres. The exposure time and temperature for the nanoparticles to attach to the material surface were optimized at 102 °C for 6 min. The entrainment of coloration intensity was confirmed when the surface was modified with sodium hydrogen carbonate at a concentration of 10 g/L, which increases the pH of the surface and the number of hydroxyl groups. Therefore, the solution containing 1 g/L nanospheres and 10 g/L sodium hydrogen carbonate was selected as the optimal composition (Figure 14b). Furthermore, surface modification confirmed that cotton retained its strength properties, indicating that nanoparticles and hydrogen carbonate do not degrade the surface. This method offers a new approach to improving color intensity and pattern clarity in inkjet printing on cotton through relatively economical and easily scalable methods [100].

**Figure 15:**
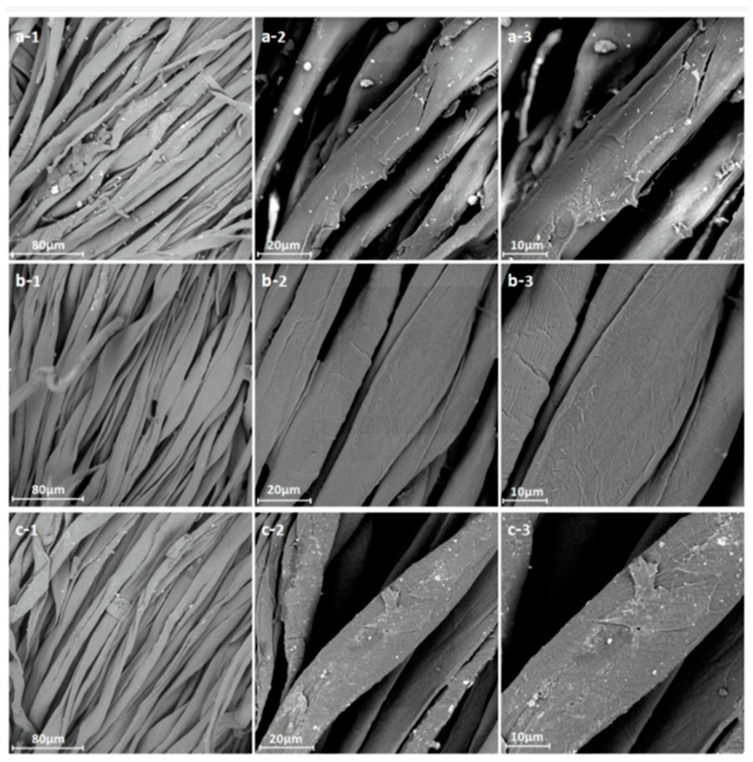
SEM images: (**a**) standard tissue, (**b**) tissue treated with a sodium percarbonate-activated system, and (**c**) tissue bleached with hydrogen peroxide; reproduced from [101].

Li et al. proposed a pre-modification method using temperature treatment of sodium percarbonate activated with tetraacetylenediamine. The material’s concentrations and exposure temperatures were optimized to enhance its hygroscopic properties and water absorption. These properties can significantly affect the coloration intensity in inkjet printing. Pre-modification was effective in cleaning the materials from defects and contaminants that may have remained on the surface after fabric production (Figure 15b). It is worth noting that standard systems based on peroxide, sodium carbonate, surfactants, and heat treatment were less effective in removing contaminants. It is important to note that the use of a lower temperature (70 °C) compared to the 90 °C used in the peroxide treatment increased the capillary effect and improved the strength properties of the material. Additionally, this composition has a bleaching property, which is significant when used in the textile industry. The use of sodium percarbonate as a low-temperature modification method can be highly relevant for treating temperature-labile materials, which are becoming more common in the textile industry [101].

### 5.3. Physicochemical Methods of Surface Modification

Physical and chemical methods can be combined to enhance image quality, durability, and droplet spreading on surfaces. This combination provides the advantage of modifying the surface both mechanically and chemically, while also imparting additional properties.

Kaimouz et al. proposed a method for pre-modifying Tencel, Tencel A100, and cotton materials using a mixture of urea, alkali, and acrylic acid as the main modifying agents, along with steam treatment of the surface of the materials. The modifying formulations were found to improve print quality compared to the original materials. During this study, we optimized the exposure time and composition ratios to enhance color intensity and inkjet print quality for each material. The results demonstrate the quantitative effect of the concentration of each substance in the modifying composition on color and dye fixation for inkjet printing [102]. This study helps personalize and improve the effectiveness of the physicochemical treatment before inkjet printing. With these results, it is possible to improve the quality of inkjet printing and increase the image’s resistance to various anthropogenic influences.

The use of ultrasound and enzymes on fabrics can result in reduced roughness, removal of contaminants, bleaching, and increased surface hydrophilicity. These effects can positively impact the inkjet printing process. Enzymatic processes, in particular, can optimize the surface condition of materials like cotton for inkjet printing.

Basto et al. optimized the parameters of the knots, including time and power, and calculated the optimal concentrations for laccase treatment. This study confirms the effectiveness of the combined effect of enzyme and knot treatment for bleaching the cotton surface [103].

In their work, Easson et al. conducted a thorough review of the use of ultrasonic treatment in combination with enzymes for cotton processing. The authors specifically examined the combined effects of cavitation bubbles generated by ultrasonic exposure and the action of proteolytic and other enzymes on fabric fibers. They presented the mechanisms of action, synergistic effects, and necessary additives, such as alkalis, buffers, and urea. The paper presents installations for UZ-treatment and optimizes the temperature parameters of this process [104].

Xu et al. proposed a modification method that uses plasma treatment and chitosan dispersion. This method increased the color depth of four-color printed blocks by up to 214%, even after surface washing. The post-printed samples were able to withstand up to 45 wash cycles without degrading image quality [105].

Hashem et al. proposed using microwave radiation in combination with a mixture of reagents as a potential step for cleaning and bleaching cotton. They used a combination of alkali and surfactants, along with microwave irradiation, for cleaning, and a combination of hydrogen peroxide, sodium silicate, and alkali, along with microwave irradiation, for bleaching. The proposed methods demonstrated high cleaning efficiency, as confirmed by the mass change data before and after treatment. Additionally, the material treatment time was only 4 min, significantly faster than traditional methods like steam treatment. Microwave radiation also shows potential for surface bleaching, with bleaching efficiency directly related to the power and time of exposure to the material. The study confirmed the feasibility of using microwave radiation for simultaneous bleaching and surface cleaning of materials [106]. This method is cost-effective, efficient, and has a short exposure time, making it a promising pretreatment option for various textile materials prior to inkjet printing.

## 6. Prospects for Inkjet Printing Applications in Textiles

Inkjet printing is a rapidly evolving and highly versatile technology, with the textile industry being one of its most dynamic application areas. In recent years, third-party applications of inkjet printing have gained increasing attention as a promising method for depositing various substances onto different surfaces. Regardless of the type of ink and substrate, a thorough understanding of their interaction is essential to achieving optimal results. A key area of research focuses on developing multifunctional modifying coatings that enhance the adhesion of ink droplets to surfaces, thereby improving print quality. Additionally, various ink modifications and additives can be utilized to optimize droplet behavior, expanding the applicability of inkjet printing across a wide range of textile materials.

The future of inkjet printing in the textile industry lies in scaling up and automating printing processes on various fabrics. The development of industrial methods for controlling the colorimetric properties of textile surfaces is crucial for standardization and large-scale production. Furthermore, the creation of novel universal adhesion promoters could further broaden the scope of inkjet printing technology within the textile sector. Another promising direction is the advancement of smart and adaptive textiles. While such materials are already being developed using inkjet printing, they have yet to achieve widespread adoption. These innovations have the potential to revolutionize the industry by introducing garments with advanced functionalities that significantly enhance everyday life.

Moreover, in response to recent global health challenges, there is a growing demand for textiles with biological properties. The increasing diversity of bacteria and viruses presents a continuous threat, making antimicrobial and antiviral textiles more critical than ever. Inkjet printing offers a viable solution for producing such functional textiles, which could help reduce the spread of infections and improve safety, particularly in densely populated urban environments. Once developed, these systems can seamlessly transition from laboratory prototypes to commercial products, integrating smoothly into industrial production processes.

By optimizing ink properties, surface characteristics, and printing conditions, inkjet printing can emerge as a versatile, cost-effective method—not only for textile dyeing but also for the development of functional fabrics with enhanced capabilities.

## 7. Conclusions

The textile industry continues to grow and evolve, driven by rising consumer demand. This expansion necessitates the adoption of advanced methods and technologies to optimize existing processes and enable the development of innovative products.

This review explores comprehensive approaches to the application of inkjet printing in the textile industry. The findings highlight that various physicochemical surface modification techniques, including polymer coatings, can enhance contrast and refine line thickness. Plasma treatment serves as an effective mechanism for controlling material wettability—whether hydrophilic or hydrophobic, depending on the type of plasma—significantly improving the colorimetric properties of inkjet-printed images. Additionally, modifying ink compositions by incorporating specific agents and optimizing rheological properties presents a viable strategy for improving dyeing quality.

Inkjet printing expands the variety of inks and substrates that can be used, unlocking new applications within the industry. This technology enables the production of textiles with superior detail and quality while offering scalability for mass production.

The textile industry stands to benefit significantly from advancements in inkjet printing, particularly in the development of multifunctional materials. While several methods exist for engineering these advanced textiles, they have yet to see widespread adoption. This review aims to outline potential strategies for optimizing inkjet printing on various textile substrates and to highlight cutting-edge technologies that can enhance fabric properties and broaden material functionalities.

## Data Availability

No data were used for the research described in the article.
